# Epithelial ovarian cancer relapsing as isolated lymph node disease: natural history and clinical outcome

**DOI:** 10.1186/1471-2407-8-367

**Published:** 2008-12-12

**Authors:** Francesco Legge, Marco Petrillo, Vincenzo Adamo, Salvatore Pisconti, Giovanni Scambia, Gabriella Ferrandina

**Affiliations:** 1 Gynecologic Oncology Unit, Department of Oncology, Catholic University of Campobasso, Italy; 2 Department of Gynecology/Obstetrics, Catholic University of Rome, Italy; 3 Medical Oncology, Department of Human Pathology, University Hospital G. Martino, Messina, Italy; 4 Oncology Unit, Taranto Hospital, Taranto, Italy

## Abstract

**Background:**

Several evidences suggested that ovarian cancer (OC) patients showing isolated lymph node recurrence (ILNR) have an indolent evolution. The aim of the study was to retrospectively review ILNR observed in our Institution over the past 11 years in order to investigate: the pattern of disease progression after the first diagnosis of ILNR, and their clinical outcome.

**Methods:**

Between September 1995 and September 2006, 523 epithelial OC were diagnosed in our centers, and 301 of these relapsed. Cases with a diagnosis of ILNR, and at least 12 months of follow up after the diagnosis of ILNR were included. Post-relapse survival (PRS) was recorded from the date of the diagnosis of ILNR to the date of death or date last seen. Survival probabilities were estimated according to the method of Kaplan and Meier and compared by the log rank test. Cox's regression model with stepwise variable selection was used to analyse the role of clinico-pathological parameters as prognostic factors for PRS.

**Results:**

Thirty-two cases were identified as ILNR (10.6% of the recurrences, and 6.1% of the OC population). Most of the patients continued to exhibit the same pattern of progression during follow up, with 75% of the patients free from peritoneal disease after 2 years from the diagnosis of ILNR. Median Post-Relapse Survival (PRS) was 37 months, and median Overall Survival (OS) was 109 months, with all patients surviving more than 2 years after the initial diagnosis. In multivariate analysis only Platinum-Free Interval (PFI) retained a prognostic role for PRS (p value = 0.033).

**Conclusion:**

ILNR represents a less aggressive pattern of OC relapse which keeps progressing in the lymph nodes in a relatively high percentage of cases. On the other hand, the occurrence of peritoneal spreading after ILNR is associated with a rapidly fatal outcome.

## Background

Ovarian cancer (OC) is the most lethal gynaecological malignancy with the vast majority of patients succumbing within 5 years from initial diagnosis [1]. A major clinical challenge in OC is the management of the relapsing disease, which is almost always fatal within an estimated median interval post-relapse-survival (PRS) of approximately 18 months [1,2]. A short duration of platinum-free-interval (PFI) has been widely reported as a crucial factor determining a short PRS [2-4]. Moreover, also the pattern of recurrence has been shown to play a role in conditioning the clinical outcomes: indeed, OC patients suffering from recurrence with a prevalent pattern of diffuse abdominal carcinomatosis exhibit an unfavourable prognosis compared to cases presenting with discrete lesions, regardless of PFI duration [2,5,6]. On the other hand, a favourable prognosis has been recently documented for patients with isolated lymph node relapses (ILNR), who experience a median PRS ranging from 26 to 114 months, according to the modality of treatment [7-10]. Several evidences suggest that lymph node disease in OC may progress in an indolent fashion; in particular i) the clinical outcome of primary OC patients staged as FIGO stage IIIC only on the basis of lymph node involvement is more favourable compared to the prognosis of patients with peritoneal FIGO stage III disease [11-14]; ii) retroperitoneal residual tumor at second-look did not seem to influence survival [15]; iii) the clinical impact of systematic lymphadenectomy at primary surgery is still a debated issue in OC [16-18], thus suggesting a minor role of extensive lymph node dissection compared to primary maximal surgical effort for intraperitonal disease. All this data suggests that tumor cells metastasizing or recurring through the lymphatic *versus *the transcoelomic route have distinct, less aggressive biological features. While ILNR could represent a peculiar setting in order to investigate whether lymphophilic OC cells tend to have a distinct, potentially indolent biologic behaviour, the natural history of disease after the diagnosis/treatment of ILNR has been not yet addressed.

We retrospectively reviewed all cases of ILNR observed in our Institution over the past 11 years in order to investigate: i) the pattern of subsequent disease progression after the first diagnosis of ILNR, and ii) the clinical outcome of patients suffering from this peculiar pattern of relapse.

## Methods

We conducted a retrospective analysis of all primary untreated invasive OC patients consecutively admitted to the Gynecologic Oncology Unit, Catholic University of Rome and Campobasso between September 1995 and September 2006. During this period 523 epithelial OC were diagnosed and treated in our centers, and 301 of these relapsed. The criteria for inclusion in the study were: 1) the initial diagnosis of epithelial invasive OC, 2) the diagnosis of isolated lymph node relapse (ILNR) documented by CT scan and clinical examination, with or without PET scan and/or histological confirmation, 3) at least 12 months of follow up after the diagnosis of ILNR. The study was approved by our institutional Ethical Committee, in compliance with the Helsinki Declaration . At time of analysis, 32 cases (10.6% of the recurrences, and 6.1% of the whole OC population) developed ILNR, and fulfilled all the inclusion criteria. Patients' characteristics at initial diagnosis are detailed in Table 1: median age was 60 years (range 45–76), and most of the patients (90.6%) were staged as FIGO stage IIIC-IV disease. At initial diagnosis all patients underwent exploratory laparotomy with cytoreductive intent: cytoreduction to residual disease < 0.5 cm was achieved in 14 (43.7%) patients, while a residual disease between 0.5 and 2 cm was obtained in 6 (18.7%) cases. Pelvic and/or aortic lymphadenectomy was performed in cases with grossly involved lymph nodes (n = 10, 31.2%), with 6 cases showing evidence of metastatic lymph node involvement. Disease was considered primarily unresectable at time of primary laparotomy in 12 cases (37.5%), and in 8 of them a second attempt of cytoreduction (interval debulking surgery) was performed given the achievement of a clinical response to chemotherapy, All patients underwent six cycles of cisplatin-based chemotherapy with (n = 26), or without (n = 6) paclitaxel. Follow up procedures were performed according to institutional guidelines [19]. For each ILNR, information on date of recurrence, pattern of disease presentation (single versus multiple lesions, site, size), Ca125 level and specific treatment were collected and entered into a computerised database. The definition of clinical relapse, i.e. physical evidence of cancer upon examination and imaging (CT scan +/- PET scan +/- histology) was used so as to be consistent with the literature which uses this end point, as the basis for the time-oriented definitions of 'sensitive/resistant/refractory' relapse [20]. The date and site (i.e. lymph node versus peritoneum versus parenchyma) of second, third and further recurrences were also recorded. The interval between first ILNR and the appearance of peritoneal disease was also calculated. The choice of ILNR treatment depended on recurrence characteristics (single or multiple sites, duration of PFI), previous treatments, patient's performance status, and changes in treatment approaches over the duration of the study. In particular, cytoreductive surgery for ILNR was preferentially attempted in patients with single/discrete number of sites of disease, PFI>6 months and, good performance status. Post-relapse survival (PRS) was recorded from the date of the diagnosis of ILNR to the date of death for disease or date last seen. Overall survival (OS) was recorded from the date of the initial diagnosis of OC to the date of death for disease or date last seen. Survival probabilities were estimated according to the method of Kaplan and Meier and compared by the log rank test [21,22]. Cox's regression model with stepwise variable selection [23] was used to analyse the role of clinico-pathological parameters as prognostic factors for PRS: only variables with p value < 0.10 in the univariate analysis were included in the multivariate analysis. Statistical analysis was carried out using SOLO (BMDP Statistical Software, Los Angeles, CA) and Statview survival tools (Abacus Concepts- Inc- Berkeley CA).

**Table 1:** Clinical/pathological characteristics of OC patients at initial diagnosis

**Characteristics**	**No. (%)**
**All cases**	32

**Age, years**	
Median (range)	60 (45–76)

**FIGO Stage**	
II	1 (3.1)
IIIB	2 (6.2)
IIIC	27 (84.4)
IV	2 (6.2)

**Histotype**	
Serous	26 (81.2)
Endometrioid	2 (6.2)
Mucinous	1 (3.1)
Undifferentiated	3 (9.4)

**Grade**	
G1-2	9 (28.1)
G3	19 (59.4)
n.a.	4 (12.5)

**Ascites**	
No	16 (50.0)
Yes	12 (37.5)
n.a.	4 (12.5)

**Ca125, IU/ml**	
Median (range)	1,121 (11-9,082)

**Peritoneal carcinomatosis**	
No	11 (34.4)
Yes	21 (65.6)

**Lymph node status (CT/Surgery)**	
Positive	19 (59.4)
Negative	13 (40.6)

**Aortic +/- pelvic lymph node node sampling**	
Yes	10 (31.2)
No	22 (68.8)

**Residual tumor at 1^**st **^surgery (cm)**	
< 0.5	14 (43.7)
0.5 – 2	6 (18.7)
> 2	12 (37.5)

**First line Chemotherapy**	
Platinum-based	6 (18.7)
Platinum/taxane-based	26 (81.3)

## Results

### Clinico-pathological characteristics of the study population at the diagnosis of ILNR

Patients' characteristics at time of diagnosis of ILNR are detailed in Table 2: median age was 62.5 years (range 46–79), and median PFI was 17.5 months (range 1–134) with 9 patients (28.1%) recurring within 12 months from the completion of first line chemotherapy. The diagnosis of ILNR was based on CT scan findings in 15 (46.9%) cases, combined PET/CT in 5 (15.6%) cases and surgical assessment in 12 (37.5%) patients. Para-aortic lymph node stations without (n = 14, 43.7%) or with pelvic lymph node stations (n = 9, 28.1%) were the most frequently involved sites. Isolated pelvic lymph node involvement was documented in 1 case, while groin, axillary, and mediastinic involvement was documented in 2, 1, and 2 cases respectively. Most of the patients (n = 19, 59.4%) showed metastatic involvement of multiple lymph node stations, and median size of the lesions was 2.2 cm (range = 1.0–6.0). In the 28 patients with known Ca125 levels at the time of recurrence, Ca125 levels were above the normal limit in 26 cases (92.9%). Nineteen (59.4%) patients were treated with chemotherapy alone, 12 (37.5%) patients were submitted to radical surgery, followed (n = 11) or not (n = 1) by chemotherapy, and one patient did not receive any treatment because of the long PFI (8 years), and the site of recurrence (mediastinum), which was judged to be at higher risk for operative complications.

**Table 2:** Clinical/pathological characteristics of OC patients at diagnosis of ILNR

**Characteristics**	**No (%)**
**Age, years**	
Median (range)	62.5 (46–79)

**PFI, months**	
Median (range)	17.5 (1–134)

**Diagnosis of 1^**st **^recurrence**	
CT scan	15 (46.9)
CT+PET scan	5 (15.6)
Surgery+CT scan	7 (21.9)
Surgery+CT+PET scan	5 (15.6)

**Site of 1^**st **^ILNR**	
Para-aortic	14 (43.7)
Pelvic + Para-aortic	9 (28.1)
Pelvic	1 (3.1)
Groin	2 (6.2)
Axillary	1 (3.1)
Mediastinum	2 (6.2)
Other sites/combination	3 (9.4)

**Number of lymph node recurrences**	
Single	13 (40.6)
Multiple	19 (59.4)

**Size of the largest lesion, cm**	
**Median (range)**	2.2 (1.0–6.0)

**Ca125 IU/ml**	
**Median (range)**	125 (8–1257)

**Treatment of 1^**st **^ILNR**	
Chemotherapy	19 (59.4)
Surgery + chemotherapy	11 (34.4)
Surgery	1 (3.1)
Not treated	1 (3.1)

### Natural history of ILNR

**Figure 1:**
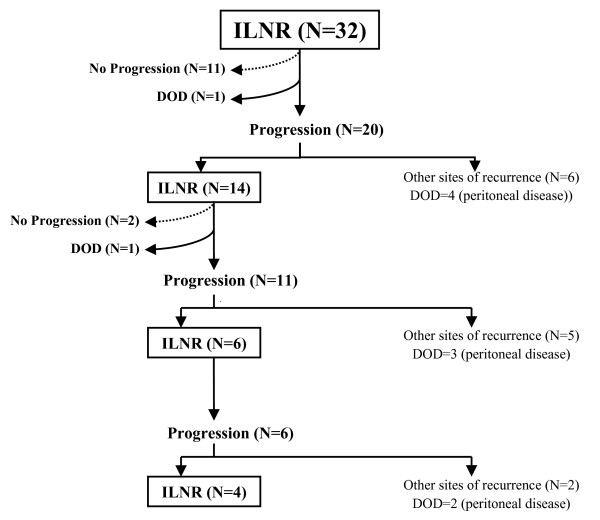
Flow chart of the events in our patient population (DOD = dead of disease).

The median follow up from the initial diagnosis of OC was 49.5 months (range = 23–149), while the median follow up from the diagnosis of ILNR was 22.5 months (range = 7–96). A flow-chart of the evolution of patients since the diagnosis of ILNR is shown in Figure 1. One patient experienced death of disease, while further progression was observed in 20 (62.5%) cases after a median interval of 12 months (range = 4–55) from the diagnosis of ILNR: 14 of these (70%) again progressed as ILNR, while 6 cases presented other sites of disease involvement, including peritoneal carcinomatosis (n = 4), lymph nodes plus peritoneum plus liver in one case, and lymph nodes plus brain in the remaining one. Of 14 cases who had progressed as ILNR, 1 experienced death of disease, and 11 showed further progression: in particular, 6 cases (54.5%) again progressed as ILNR, while 5 cases presented intra-abdominal progression. Of 6 cases who kept progressing at lymph node level, 4 (66.7%) still experienced further progression as ILNR, while 2 progressed in the abdomen. At the time of the analysis 9 (28.1%) patients developed peritoneal disease and 75% of the patients were free from peritoneal disease at 24 months from the first diagnosis of ILNR.

### Clinical outcomes of patients with ILNR

**Figure 2:**
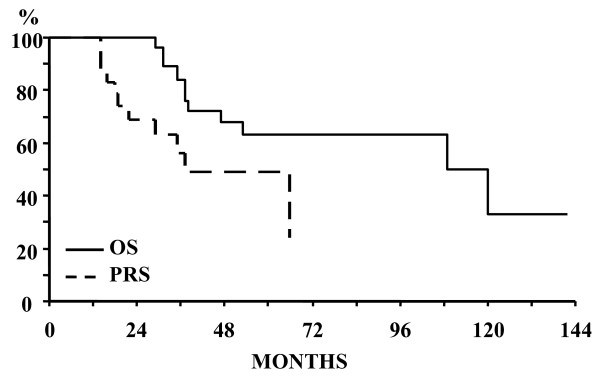
Overall Survival (OS), and Post-Relapse Survival (PRS) of the whole study population.

As of November 2007, all patients were available for survival analysis. As shown in Figure 2, median survival after ILNR (PRS) was 37 months, with 69% of the patients surviving more than 2 years after ILNR; median OS from the diagnosis of OC was 109 months, with all patients surviving more than 2 years after the initial diagnosis of OC.

Overall, at time of analysis, 11 deaths were observed: 2 deaths were documented in patients progressing as ILNR, while 9 deaths occurred in the group of patients progressing with intra-abdominal disease at any time during follow up. In particular, in the latter group of patients, the median survival after the diagnosis of peritoneal disease was 4 months (range = 2–35).

### Analysis of factors predictive of survival after ILNR

**Figure 3:**
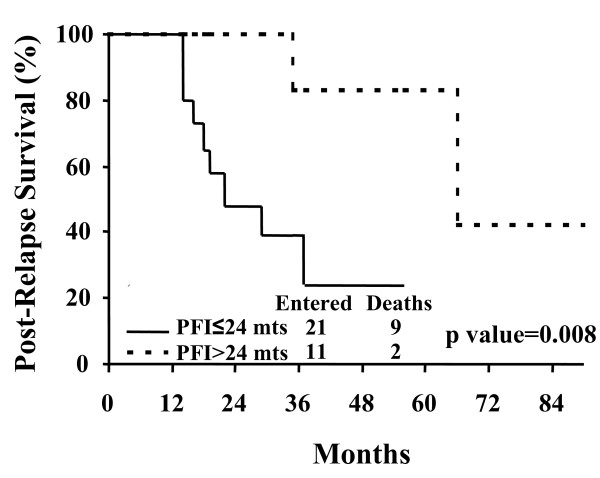
Post-Relapse Survival (PRS) according to Platinum-free-interval (PFI) (≤ 24 months versus >24 months).

Among the clinico-pathological characteristics registered at initial diagnosis and also at the time of ILNR documentation, a prolonged time to the occurrence of ILNR was associated with a better prognosis, with a median PRS of 22 months in patients with a PFI within 24 months versus a median PRS of 60 months in patients with a PFI>24 months (p value = 0.008) (Figure 3). Moreover, a more favourable outcome was documented in the 12 patients submitted to cytoreductive surgery for ILNR (1 death, median PRS = n.r.) compared to the 20 patients who did not undergo surgery (10 deaths, median PRS = 31 months), although the statistical significance was of borderline value (p value = 0.07). In this context, it is worth noting that there was no difference in the percentages of cases with intra-abdominal progression in patients who received surgical versus medical treatment of the first ILNR (16.7% versus 21.0%, respectively, p value = 0.6). Similarly, a trend to a better prognosis was found for patients younger than 60 years at initial diagnosis (p value = 0.07). Although the subgroups were too small to gain statistical significance, we failed to find any association between the site of ILNR recurrence and prognosis (data not shown).

Notwithstanding the limited number of patients and events could restrict the factors which can be included in the Cox's regression model, in the multivariate analysis assessing the impact of several factors potentially influencing the outcome of this population (age at diagnosis, PFI duration, surgical cytoreduction of ILNR), only PFI retained an independent prognostic role for PRS (p value = 0.033; χ^2 ^of the model = 7.45, p value of the model = 0.006).

## Discussion

Isolated lymph node recurrence (ILNR) of ovarian cancer (OC) represents a rare but not exceptional pattern of disease which has recently gained much attention in the literature [7-10]. In accordance with previously reported data [7], ILNR occurred in about 6% of our OC patients representing approximately 10% of the overall recurrences. The rate of ILNR in our population, as well as in other studies not performing systematic lymphadenectomy at initial surgery [7], was similar to the results reported in series including patients submitted to systematic pelvic/para-aortic lymphadenectomy [15-17,24]. Moreover, in two randomised trials comparing systematic lymphadenectomy versus resection of only bulky lymph nodes no difference in the percentage of lymph node recurrences was documented [16,17].

In our series most of the patients exceed 9-years survival from the initial diagnosis, thus confirming the favourable prognosis of recurrent OC patients suffering from ILNR [7-10]: this finding becomes even more relevant considering that compared to previous studies (Table 3), our patients were unselected for the duration of PFI, one of the major determinants of prognosis, and were submitted to cytoreduction of recurrence in only 37.5% of the cases.

**Table 3:** Clinical/pathological features at initial diagnosis and outcome details of OC patients recurring as ILNR: data from the available literature

**Author**	**Blanchard et al. **[7]	**Benedetti Panici et al. **[8]	**Santillan et al. **[9]	**Uzan et al. **[10]	**Current study**
**ILNR**					
**- N° of cases**	27	19	25	12	32
**- % of OC recurrences**	n.r.	n.r.	n.r.	n.r.	10.6
**- % of OC**	4.2	n.r.	n.r.	n.r.	6.1

**Stage III-IV**	67%	60%	72%	50%	94%

**RT at first surgery >2 cm**	n.r.	<5%^a^	8%	8.3%	37.5%

**Poor differentiated**	n.r.	60%	100%	n.r.	68%

**Systematic lymphadenectomy at first surgery**	0	0	0	0	0

**Complete response after Initial treatment**	59.3%	n.r.	100%	n.r.	65.6%

**Histological diagnosis of ILNR**	63%	100%	100%	100%	37.5%

**Cases with CA125 levels**					

**>35 U/ml at the time of ILNR**	92%	60%	50%	100%	93%

**PFI, months Median (range)**	26 (1–159)	14 (7–84)	16 (6–40)	21 (6–72)	17.5 (1–130)

**Site of ILNR**					
**-Aortic N° (%)**	15 (55.5%)	30 (75%)^b^	16 (64%)	7 (58.3%)	23 (71.9%) ^b^
**-Pelvic N° (%)**	4 (14.8%)	10 (25%)	4 (16%)	5 (41.7%)	1 (3.1%)
**-Other N° (%)**	14 (51.8%)	n.r.	6 (24%)	2 (16.7%)	8 (25.0%)

**Secondary cytoreduction**	29.6%	72.5%	100%	100%	37.5%

**Median follow up duration from ILNR diagnosis (mts)**	n.r.	26	19	50	19.5

**Median PRS (mts)**	26	>60	37	>60	37

**Median OS (mts)**	68	n.r.	61	114	109

**Prognostic factors influencing PRS**	None	-Secondary cytoreduction	None	n.r.	-PFI>24 mts -Secondary surgery

n.r. = not reported

^a ^Residual tumor > 1 cm

^b ^Para-aortic with or without pelvic lymph node involvement

The favourable prognosis of patients with ILNR is unlikely to be related to the serendipitous selection of patients with good clinico-pathological features at initial diagnosis: indeed, in our series 90.6% of cases were stage IIIc-IV disease, and 37.5% were not optimally cytoreduced at primary surgery. Conversely, the median duration of PFI was longer compared to that usually reported in the overall OC population [2], thus suggesting that the late occurrence of lymph node relapse is intrinsically related to its more indolent behaviour.

We also showed for the first time that cases presenting with ILNR keep progressing at lymph node level in a relatively high percentage of cases: after the first ILNR, progression of disease was documented in 20 patients and in 70% of them disease was still limited to lymph node stations; notably, at time of final analysis, 4/20 cases (25.0%) still continued to show disease only at lymph node levels suggesting the persistence of long lasting, less aggressive lymphophilic features. Indeed, after 2 years from the diagnosis of ILNR 75% of the patients were free from peritoneal disease.

In the group of patients with ILNR we observed only 2 deaths of disease: one patient presented bulky lympho-adenopathies infiltrating the cava vein and lumbar vertebrae, thus hampering the possibility to perform retroperitoneal cytoreduction; the other case was a 82 years old woman who was not operated on because of the presence of several co-morbidities judged to carry out a very high risk for operative and postoperative morbidity. On the other hand, although the development of peritoneal tumor spreading was shown to be relatively belated, it can occur any time after the first ILNR progression and it is rapidly aggressive: at time of analysis all cases progressing in the peritoneum had experienced death of disease.

In this context, a more in depth understanding of the biology of the metastatic process in both primary and recurrent OC, through the characterization of the molecular pathways regulating the pattern of spread to peritoneal or lymph node stations, would be relevant: indeed, several factors associated with anti-apoptotic, pro-angiogenic and pro-invasiveness functions as well as with drug resistance have been reported to play a role in peritoneal diffusion [25], while there is scanty data, if any, concerning the biology of lymph node route of cancer spreading. We can hypothesize that the relatively indolent behaviour shown by ILNR can be related to: i) an intrinsic nature of OC cells, characterized by low proliferation rates and inability to initially express molecules associated with peritoneal spreading; ii) the peculiar microenvironment of the lymph node, where T cells and cytokines may keep tumor cells in a dormant state thus containing tumor spread. The analysis of the immunological characteristics of the lymph node's cell population, together with the pathological features of the tumor infiltration (e.g. microscopic with T cell infiltration *versus *massive neoplastic invasion) and, the molecular characterization of the tumor cells through genomic/proteomic techniques, could be particularly useful to understand the different clinical behaviour associated with distinct patterns of tumor dissemination.

Finally, we showed that among the features potentially affecting the clinical outcome after ILNR, a long duration of PFI as well as the surgical removal of ILNR, were associated with a more favourable prognosis; this data is not surprising considering that platinum sensitivity represents the major determinant of prognosis in recurrent OC patients, together with surgical cytoreduction of the recurrent lesion in selected clinical setting [5,6]. It could be argued that in our series the diagnosis of ILNR was surgically confirmed only in 37.5% of the cases, thus leading to a potential underestimation of the presence of peritoneal lesions, as suggested by Bristow et al. [26], who documented the presence of occult intraperitoneal disease in 21.4% of ILNR even when PET/CT scan techniques were used. However, our data shows similar percentages of intra-abdominal progression in patients who had surgical versus medical treatment of the first ILNR, thus suggesting that the favourable role of cytoreductive surgery is more reasonably related to removal of the disease rather than to the selection of cases without peritoneal seeding.

Although in multivariate analysis only the long duration of PFI was shown to maintain its favourable prognostic role for PRS, the usefulness of surgical exploration of patients with suspected ILNR should be not underestimated, and complete surgical resection of lymph node stations should be attempted in the absence of diffuse peritoneal disease, given the overall localized and slowly growing features of lymph node disease.

## Conclusion

In conclusion, although these findings need to be confirmed in a larger series, ILNR seems to represent a less aggressive pattern of disease relapse which keeps progressing as ILNR in a discrete proportion of cases. On the other hand, the relatively belated occurrence of peritoneal spreading may occur any time after the first ILNR documentation and is necessarily associated with a rapidly fatal outcome.

The multiparametric molecular characterization of peritoneal and lymph node disease, in both primary and recurrent OC, would possibly provide useful information in the future.

## Competing interests

The authors declare that they have no competing interests.

## Authors' contributions

FL contributed to the conception and design of the study, statistical analysis and interpretation of the results, drafting of the final manuscript

MP contributed to the data collection and table and figures preparation

VA contributed to the data collection

SP contributed to the data collection

GS contributed to the conception, design of the study and interpretation of the results.

GF contributed to the conception and design of the study, analysis and interpretation of the results, drafting of the final manuscript.

All authors read and approved the final paper.

## Pre-publication history

The pre-publication history for this paper can be accessed here:


